# Incidence and factors associated with Herpes Zoster infection in kidney transplant recipients, a recent epidemiological study

**DOI:** 10.3389/ti.2026.14920

**Published:** 2026-06-10

**Authors:** Benjamin Taton, Pierre Pfirmann, Isabelle Garrigue, Karine Moreau, Marine Novion, Frédéric Jambon, Pierre Merville, Lionel Couzi, Hannah Kaminski

**Affiliations:** 1 Department of Nephrology, Transplantation, Dialysis and Apheresis, Bordeaux University Hospital, Bordeaux, France; 2 University Bordeaux, Fundamental Microbiology and Pathogenicity (MFP), UMR 5234 and Laboratory of Virology, Bordeaux University Hospital, Bordeaux, France; 3 University Bordeaux, CNRS, INSERM, ImmunoConcEpT, UMR 5164, ERL U1303, Bordeaux, France

**Keywords:** epidemiology, herpes zoster, incidence, kidney transplant recipients, vaccine

Dear Editors,

We want to address a letter about incidence and factors associated with herpes zoster infection in Kidney transplant recipients through a recent epidemiological study.

Herpes zoster (HZ), resulting from varicella-zoster virus reactivation, occurs more frequently in immunocompromised patients, including kidney transplant recipients (KTRs). However, contemporary epidemiological data focusing on age-specific incidence and risk factors in homogeneous cohorts of KTRs remain limited [1–6].

We conducted a monocentric observational cohort study including all adult patients who underwent kidney transplantation at our center between 2004 and 2015, had a functioning graft at day 30, and received a standard immunosuppressive regimen based on a calcineurin inhibitor combined with mycophenolate or everolimus. Patients with multi-organ transplantation or HIV infection were excluded. Follow-up extended until January 2017, death, or graft failure. Cytomegalovirus prophylaxis consisted of valganciclovir for 3 or 6 months according to donor and recipient serostatus, or a preemptive strategy.

Herpes zoster cases were identified through a systematic keyword search of shared electronic medical records and validated by expert review based on clinical description; virological confirmation was required only for disseminated or organ-invasive disease. Incidence rates were calculated overall and by age group, with 95% confidence intervals obtained by bootstrap resampling. Cumulative incidence was estimated using the Aalen–Johansen method, accounting for death and graft failure as competing events. Risk factors for HZ were evaluated using cause-specific Cox proportional hazards models, with valganciclovir exposure modeled as a time-varying covariate. Variables associated with HZ at p < 0.20 in univariable analyses were included in multivariable models, followed by backward elimination retaining variables with p < 0.05, after colinearity checking.

A total of 1,101 KTRs were included, with a median follow-up of 5.6 [3.3–9.2] years, representing 6092 patient-years. Eighty-nine patients experienced at least one episode of HZ, yielding an incidence rate of 14.5 per 1,000 patient-years (95% CI: 11.5–17.6). The median time to HZ onset was 5.3 years post-transplantation Among the 99 zoster herpes, 20 were severe (22.5%; ophtalmic or neurological involvment, post-herpetic neuralgia, disseminated VZV, tissue invasive/organ disease), 53 non severe (59.5%); and 16 undetermined (18%).

Recipients who developed HZ were older at transplantation (p = 0.042), had a higher number of HLA class II mismatches (p = 3.2 × 10^−4^), and were more likely to receive maintenance corticosteroids (p = 0.004). When stratified by age quartiles, cumulative incidence analyses showed a significantly higher risk of HZ in recipients aged >44 years at transplantation compared with younger patients (p = 0.0045).

In the multivariable model, older age (HR 1.30 per decade, 95% CI 1.10–1.50; p = 0.008), induction with rabbit anti-thymocyte globulin (HR 1.70, 95% CI 1.10–2.60; p = 0.02), and higher HLA class II mismatch (HR 1.40, 95% CI 1.20–1.70; p = 6 × 10^−4^) remained independently associated with HZ occurrence, whereas valganciclovir exposure was not.

In addition, kidney transplant recipients experienced a substantially increased risk of herpes zoster across all age groups, with incidence rates in younger recipients comparable to those observed in elderly individuals in the general population (Figure 1). In all age ranges the incidence rate of HZ was much higher than in the general population (geometric mean of incidence rate ratios: 3.6). It is worth noting that even below 25 years of age at the time of transplantation the incidence rate of HZ was 6.33/1,000 patient-years [0–16.3], approximately at the same level as in the general population between 55 and 65-year (5.77 [4.97–6.97] [7]. These findings provide robust epidemiological support for systematic use of the recombinant zoster vaccine in all adult KTRs irrespective of age. As the vaccine has a good immunogenicity in immunocompromised patients [8], they support broader implementation and reimbursement strategies in transplant populations, for futures European Guidelines.

**FIGURE 1:**
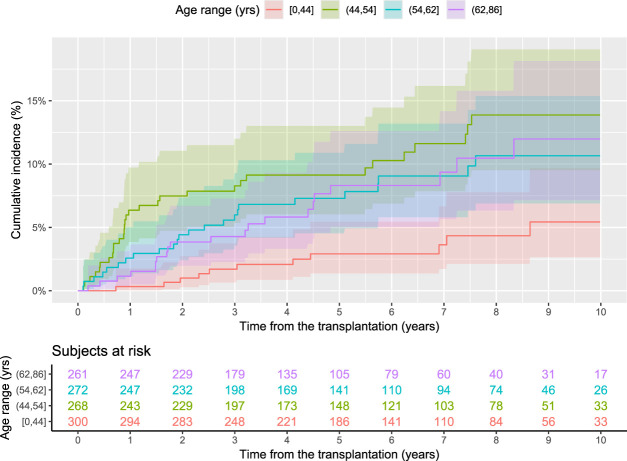
Incidence rate of Herpes Zoster of our cohort vs. Pinchinat et al. (DOI: 10.1186/1471-2334-13-170) across ages. Bar and error plot comparing incidence rates of cases per one thousand patient-years for kidney transplant recipients and general population by age group, showing higher rates for transplant recipients in most age ranges, with rate ratios listed below the chart.

## Data Availability

The original contributions presented in the study are included in the article/supplementary material, further inquiries can be directed to the corresponding author.

## References

[1] Hurley JK Greenslade T Lewy PR Ahmadian Y Firlit C . Varicella-zoster infections in pediatric renal transplant recipients. Arch Surg Chic Ill (1960) 115(6):751–2. 10.1001/archsurg.1980.01380060049012 6992736

[2] Hogewoning AA Goettsch W van Loveren H de Fijter JW Vermeer BJ Bouwes Bavinck JN . Skin infections in renal transplant recipients. Clin Transpl (2001) 15(1):32–8. 10.1034/j.1399-0012.2001.150106.x 11168313

[3] Gourishankar S McDermid JC Jhangri GS Preiksaitis JK . Herpes zoster infection following solid organ transplantation: incidence, risk factors and outcomes in the current immunosuppressive era. Am J Transpl (2004) 4(1):108–15. 10.1046/j.1600-6143.2003.00287.x 14678041

[4] Martin-Gandul C Stampf S Héquet D Mueller NJ Cusini A van Delden C Preventive strategies against cytomegalovirus and incidence of α-Herpesvirus infections in solid organ transplant recipients: a nationwide cohort study. Am J Transpl (2017) 17(7):1813–22. 10.1111/ajt.14192 28039960

[5] Fernández-Ruiz M Origüen J Lora D López-Medrano F González E Polanco N Herpes zoster in kidney transplant recipients: protective effect of anti-cytomegalovirus prophylaxis and natural killer cell count. A single-center cohort study. Transpl Int (2018) 31(2):187–97. 10.1111/tri.13076 28940695

[6] Sarı N Erol Ç Yanık Yalçın T Kurt Azap Ö Arslan H Karakaya E Herpes zoster infections in solid-organ transplant recipients. Exp Clin Transpl (2023) 21(9):764–71. 10.6002/ect.2023.0185 37885293

[7] Gonzalez CS Sarazin M Turbelin C Lasserre A Pelat C Bonmarin I Herpes zoster: burden of disease in France. Vaccine (2010) 28(50):7933–8. 10.1016/j.vaccine.2010.09.074 20946861

[8] Vink P Ramon Torrell JM Sanchez Fructuoso A Kim SJ Kim SI Zaltzman J Immunogenicity and safety of the adjuvanted recombinant zoster vaccine in chronically immunosuppressed adults following renal transplant: a phase 3, randomized clinical trial. Clin Infect Dis (2020) 70(2):181–90. 10.1093/cid/ciz177 30843046 PMC6938982

